# Current Trends in Studies of Ancient Diseases

**DOI:** 10.1155/2019/1472632

**Published:** 2019-01-22

**Authors:** Dong Hoon Shin, Raffaella Bianucci, Robert D. Loynes, Hisashi Fujita, Myeung Ju Kim

**Affiliations:** ^1^Seoul National University, Seoul, Republic of Korea; ^2^The University of Warwick, Coventry, UK; ^3^University of Manchester, Manchester, UK; ^4^Niigata College of Nursing, Niigata, Japan; ^5^Dankook University, Cheonan, Republic of Korea

For decades, paleopathology, the science that investigates diseases and related conditions by examination of skeletal and soft-tissue remains, has allowed scholars to reconstruct historical populations both demographically and epidemiologically. Thanks to the recent advances in paleogenetics, knowledge on ancient human migrations, diets, and pathogens has been greatly augmented. More recently, the systematic pathographic approach to the study of ancient diseases has added further to the accumulated data on how ailments were diagnosed and treated in different social-cultural contexts. By combining the pathological evidence on skeletal and mummified remains with the analysis of ancient texts and literary sources, a holistic approach to the study of past morbidity conditions and related treatments can be applied. This special issue aims to provide examples of these new trends.

G. D. Al-Khafif et al. provided the first evidence of nonfalciparum malaria in the skeletal remains of Old Kingdom Egyptians from the Giza skeletal collection. Resorting to an immunochromatographic assay (RDT), they detected aldolase, the pan-malarial antigen, in 56% of analyzed individuals, and found no significant differences between wealthy officials and workers. As described in ancient texts, the annual Nile flooding tended to affect increased numbers of mosquito breeding sites; starting with the Dynastic Period, this trend was accelerated by the construction of irrigation systems. Archaeological and textual evidence indicates that Ancient Egyptians protected themselves from the Anopheles mosquitoes; for instance, the bed of 4th Dynasty Queen Hetepheres I, the wife of King Snefru who ruled Egypt between c 2613 and 2589 BC, most likely had been designed to bear a protective bednet. Similarly, the use of herbal remedies for malaria (possibly garlic-based remedies) was reported by the Greek historian Herodotus. G. D. Al-Khafif et al. also uncovered evidence that, independently of social status, both high officials and workers on Giza's plain were exposed to the same threat of contracting malaria. Whereas no information on the use of protections against malaria is available for the Giza site, there is evidence that medical treatments, including surgery (i.e., limb amputation), were equally available to members of higher and lower social groups.

Some studies in this special issue had dealt with East Asian cases of ancient skeletal disease. T. Tsurumoto et al. identified a metastatic bone tumor, possibly a prostatic cancer, in a 5th-6th century Japanese skeleton. The skeleton displayed multiple osteoblastic bone lesions with a scapular sunburst appearance, cortical bone thickening with periosteal reaction, and osteosclerotic changes to the trabecular structure of cancellous bones. Y.-S. Kim et al. comprehensively analyzed lumbosacral defects and investigated the occurrence of spina bifida occulta (SBO), lumbosacral transitional vertebrae (LSTV), and spondylolysis in 16th- 18th century skeletons exhumed from Korean Joseon graves. These datasets shed new light on the ways of living and physical conditions of ancient East Asian populations.

As highlighted in this special issue, other multidisciplinary studies also have been conducted. L. Guedes et al. diagnosed probable syphilis cases in human remains unearthed in Brazil. Historical sources had documented the occurrence of endemic treponemal infections in Rio de Janeiro between the 17th and 19th centuries. Although no morphological evidence of treponematoses was present in the analyzed samples (n = 25) obtained at the Nossa Senhora do Carmo Church (17th–19th century) and Praça XV Cemetery (18th–19th century) sites, ancient DNA analyses provided, in accordance with the historical sources, evidence of cases of probable syphilis.

Perusal of documentary sources dating to the Joseon Dynasty (17th–19th centuries) coupled with current scientific data on the evolutionary history of* P. vivax* in East Asia allowed D. H. Shin et al. to reconstruct the history of malaria in Korea. The authors highlighted how the life habits of the Joseon people might have facilitated the transmission of the protozoan, thus unwittingly contributing to the high incidence of malaria in Korea at that time.

Over the decades, mummy studies have expanded in reconstructing multifaceted databases on living conditions, pathologies, and possible causes of death in different spatiotemporal contexts. D. H. Shin et al. summarized the outcomes of studies performed in Korea and China in order to provide mummy experts with hitherto largely unknown facts on East Asian mummies. In so doing, they showed that the Korean and Chinese mummies examined share a common cultural background.

A thorough knowledge of historical backgrounds is required in order to fully assess disease burdens of past populations. The purpose of this special issue is to present new achievements in the fields of paleopathology and bioanthropology and to contextualize them in their relevant sociocultural frames.

